# Advantages of Continuous and Non-Invasive Glucose Monitoring in the Geriatric Population: A Systematic Review

**DOI:** 10.3390/jcm15093194

**Published:** 2026-04-22

**Authors:** Eric Oliviu Coșovanu, Andrei Szilagyi, Alexandra Szilagyi, Elena Teona Coșovanu, Luiza Elena Corneanu, Mara Sînziana Sîngeap, Bogdan Ionel Tamba, Lucian Hrițcu, Ovidiu Rusalim Petriș

**Affiliations:** 1 “Grigore T. Popa” University of Medicine and Pharmacy, 16 Universitatii Street, 700115 Iasi, Romania; cosovanu_eric-oliviu@d.umfiasi.ro (E.O.C.); alexandra.szilagyi@umfiasi.ro (A.S.); cosovanu_elena-teona@d.umfiasi.ro (E.T.C.); luiza-elena.corneanu@umfiasi.ro (L.E.C.); singeap.mara-sinziana@d.umfiasi.ro (M.S.S.); ovidiu.petris@umfiasi.ro (O.R.P.); 2 Department of Biology, Faculty of Biology, Alexandru Ioan Cuza University of Iasi, 700506 Iasi, Romania; hritcu@uaic.ro; 3 Advanced Research and Development Center for Experimental Medicine “Prof. Ostin C. Mungiu”—CEMEX, “Grigore T. Popa” University of Medicine and Pharmacy, 700115 Iasi, Romania; bogdan.tamba@umfiasi.ro; 4 Department of Medical Procedures and First Aid Skills, “Grigore T. Popa” University of Medicine and Pharmacy, 700115 Iasi, Romania; 5 Department of Pharmacology, Clinical Pharmacology and Algesiology, “Grigore T. Popa” University of Medicine and Pharmacy, 700115 Iasi, Romania

**Keywords:** continuous glucose monitoring (CGM), diabetes mellitus, older adults, glycemic control, hypoglycemia, time in range (TIR), non-invasive glucose monitoring

## Abstract

**Background:** The global aging population faces an increasing prevalence of type 2 diabetes mellitus (T2DM), often complicated by frailty, cognitive decline, and impaired manual dexterity. These factors make glucose self-monitoring particularly challenging. Minimally invasive glucose monitoring methods, particularly continuous glucose monitoring (CGM) as well as emerging non-invasive glucose monitoring technologies offer potential solutions, but remain insufficiently evaluated in older adults. **Objective:** To systematically review and synthesize available evidence on the advantages of continuous glucose monitoring (CGM) and non-invasive glucose monitoring methods in older adults aged ≥65 years, focusing on clinical efficacy, usability, adherence, and existing knowledge gaps. **Methods:** A systematic literature search was conducted across PubMed, Scopus, and Web of Science, including studies from 2020 to 2025. Eligible studies included participants aged ≥65 years and evaluated the clinical performance of CGM or other minimally invasive or non-invasive glucose monitoring technologies. The PRISMA framework guided screening and selection. Risk of bias was assessed using RoB 2 and ROBINS-I tools. Due to substantial heterogeneity among study designs and reported outcomes, a narrative synthesis approach was adopted. **Results**: A total of 426 records were identified, of which 13 met the predefined eligibility criteria after full-text screening. After risk of bias assessment, one study was excluded, resulting in 12 studies included in the final synthesis. No eligible studies evaluating completely non-invasive glucose monitoring technologies were identified, highlighting a significant research gap in this area specifically for older adults. CGM was associated with improved glycemic control, reduced hypoglycemia, and increased time in range among older adults. Usability was generally high, particularly with newer, user-friendly devices. **Conclusions:** CGM is associated with improved glycemic outcomes and favorable usability in adults aged ≥65 years. However, a significant gap exists in research on non-invasive glucose monitoring technologies in this population. Future studies should address the accuracy, feasibility, and usability of non-invasive glucose monitoring devices, while accounting for the physiological and behavioral complexities associated with aging.

## 1. Introduction

As the global population ages, the prevalence of type 2 diabetes mellitus (T2DM) among older adults continues to rise, estimated at 23.7% [1], posing significant challenges to health systems and individual well-being. Frailty is a significant concern among older adults aged ≥65 years, particularly in individuals with diabetes, with reported prevalence rates ranging from 53.8% to 61.8% across different populations [2,3]. Frailty is associated with a worse prognosis, including increased mortality, hospitalization, and risk of hypoglycemia [4].

At the same time, cognitive impairment among older adults aged ≥65 years is a significant public health concern, characterized by a decline in mental abilities such as memory and attention. In China, the prevalence of cognitive impairment among older adults was 44.04%, with higher rates in females and those with lower education levels [5]. In a hospital-based study, 41.2% of adults aged ≥65 years were found to have cognitive impairment [6].

Cognitive decline in older adults is associated with impaired hand dexterity and reduced bimanual coordination, which may compromise their ability to perform routine functional tasks. Tasks involving executive functions and attention are particularly affected, indicating a strong link between cognitive function and manual dexterity [7]. Older adults show a decline in force release performance during precision grip tasks, which may be due to sensory deficits and changes in central nervous system circuits [8]. Age-related decreases in motor and sensory nerve conduction velocities have been observed, with reduced motor nerve conduction velocity being directly associated with decreased fingertip dexterity [9].

As individuals age, physiological changes such as declining renal function increase susceptibility to both hyperglycemia and hypoglycemia, particularly during surgical procedures or periods of stress [10]. Hypoglycemic episodes may also exacerbate previously mild physical and cognitive impairments [11]. These factors necessitate careful monitoring and individualized glycemic control strategies to prevent complications and improve quality of life.

Given the importance of simplifying diabetes management to reduce treatment burden and enhance adherence among older adults, particular attention should be paid to how glycemia is measured and monitored in this population [12]. Non-invasive and minimally invasive methods address these challenges by providing safe, pain-free, user-friendly, and continuous monitoring solutions. Non-invasive methods eliminate the discomfort associated with finger pricking, thereby improving patient adherence and self-management. In parallel, minimally invasive CGM devices have been shown to reduce glycemic variability and hypoglycemia in older adults, contributing to improved overall glycemic control [13].

The objective of this systematic review is to synthesize the available evidence regarding the advantages of CGM and non-invasive glucose monitoring methods specifically in older adults aged ≥65 years. This review evaluates the impact and potential benefits of these technologies on clinical outcomes, patient adherence, quality of life, and the use of healthcare services compared with traditional methods of glycemic monitoring. By highlighting current findings and identifying knowledge gaps, this review aims to inform clinical decision-making, support the integration of advanced glucose monitoring technologies in older populations, and underscore the importance of addressing reimbursement policies that may affect access.

## 2. Materials and Methods

We conducted a systematic literature search to identify relevant studies for inclusion in this review. The review was conducted and reported in accordance with the Preferred Reporting Items for Systematic Reviews and Meta-Analyses (PRISMA) 2020 guidelines. The completed PRISMA 2020 checklist is provided as Supplementary Material (Table S1). The protocol was registered in the PROSPERO database (CRD420251081838). Searches were performed in PubMed/MEDLINE, Scopus, and Web of Science. The initial search was conducted on 15 May 2025 and updated on 20 January 2026. The final search date was therefore 20 January 2026. The search covered studies published between 1 January 2020 and 31 December 2025.

We used a combination of Medical Subject Headings (MeSH) and free-text terms relevant to the research topic. The full search strategy for each database is provided in Appendix A.

The total number of records identified prior to deduplication was as follows: PubMed (*n* = 6724), Scopus (*n* = 10741), and Web of Science (*n* = 443).

The search strategy was intentionally designed to capture both continuous glucose monitoring (CGM) and fully non-invasive glucose monitoring technologies. Specific terms related to non-invasive monitoring approaches (e.g., spectroscopy-based methods, transdermal sensing, and alternative biological fluids) were included to ensure comprehensive coverage. The absence of eligible studies on non-invasive glucose monitoring in older adults was therefore identified as a result of the completed systematic search process rather than a limitation of the search strategy.

We screened all retrieved articles in two stages: first by title and abstract, followed by full-text review. Studies were eligible for inclusion if they met all of the following predefined criteria: (1) evaluated continuous glucose monitoring (CGM) or non-invasive glucose monitoring (NIGM) methods; (2) included participants aged ≥65 years, populations in which at least 50% of participants were aged ≥65 years, or reported subgroup analyses specifically for individuals aged ≥65 years; and (3) were primary research studies (including randomized controlled trials, observational studies, cohort studies, validation, feasibility, pilot, or usability studies) published in English between 2020 and 2025.

We considered studies evaluating both minimally invasive and non-invasive glucose monitoring technologies, including optical methods (e.g., Raman or near-infrared spectroscopy), reverse iontophoresis, electromagnetic sensing, transdermal sensors, and devices using alternative biological fluids such as sweat, saliva, or interstitial fluid. Comparator methods could include traditional invasive glucose monitoring; however, studies without comparators, such as single-arm feasibility or performance studies, were also considered eligible.

These eligibility criteria were defined a priori and applied consistently during the screening process. Studies that did not clearly meet the age-related inclusion criteria were excluded to minimize selection bias and ensure methodological rigor.

Studies were excluded if they focused exclusively on populations under 65 years without age-stratified data or involved animal or in vitro experiments. We also excluded studies evaluating only invasive glucose monitoring devices unless used as comparators, studies lacking outcomes related to usability, accuracy, adherence, or clinical impact, as well as technical validation studies without human subjects. Additionally, case reports or case series with fewer than five patients, non-English publications, and studies without accessible full text were excluded.

After removing duplicates, 422 unique records were screened by title and abstract. Following this initial screening stage, 29 articles were identified as potentially relevant and were retained for full-text review. After full-text assessment, 13 studies met the predefined eligibility criteria and underwent methodological assessment.

Subsequently, we conducted a risk of bias assessment using the Cochrane Risk of Bias 2 (RoB 2) tool for randomized controlled trials and the Risk Of Bias In Non-randomized Studies of Interventions (ROBINS-I) tool for observational studies. Based on this assessment, one study was excluded due to critical risk of bias, resulting in a final sample of 12 studies included in the final synthesis. Detailed justifications for this exclusion, along with individual risk of bias assessments, are provided in Appendix B.

The search strategy was designed to include both continuous glucose monitoring (CGM) and non-invasive glucose monitoring methods in the geriatric population. Despite a comprehensive search across PubMed, Scopus, and Web of Science, many studies meeting the inclusion criteria focused on CGM. This reflects a current gap in published research on non-invasive glucose monitoring techniques in older adults.

Data extraction was conducted independently by two reviewers using a pre-piloted standardized form. Extracted data included study design, population characteristics, type of monitoring technology used, primary outcomes and key findings. Discrepancies were resolved through discussion or consultation with a third reviewer.

To assess the risk of bias in the included studies, we used the Cochrane Risk of Bias 2 (RoB 2) tool for randomized controlled trials and the Risk Of Bias In Non-randomized Studies of Interventions (ROBINS-I) tool for observational and non-randomized studies. These assessments were conducted independently by two reviewers, and studies were categorized as having low, moderate, or high risk of bias based on the criteria specific to each tool.

The certainty of evidence for the main outcomes was assessed qualitatively across outcome domains, taking into account study design, risk of bias, consistency of findings, directness, and precision. Outcomes supported by randomized controlled trials with generally consistent findings were considered to provide higher certainty than those informed mainly by observational, feasibility, or pilot studies. The certainty of evidence was summarized as moderate, low, or not assessable, as appropriate for each outcome domain.

Given the substantial heterogeneity across study populations, diabetes types, monitoring technologies, comparators, settings, and reported outcome metrics, a meta-analysis was not considered appropriate. Therefore, findings were synthesized narratively and grouped by monitoring modality and outcome domain, including glycemic control, hypoglycemia, usability/adherence, and other clinically relevant outcomes.

## 3. Results

### 3.1. Study Selection

**Figure 1:**
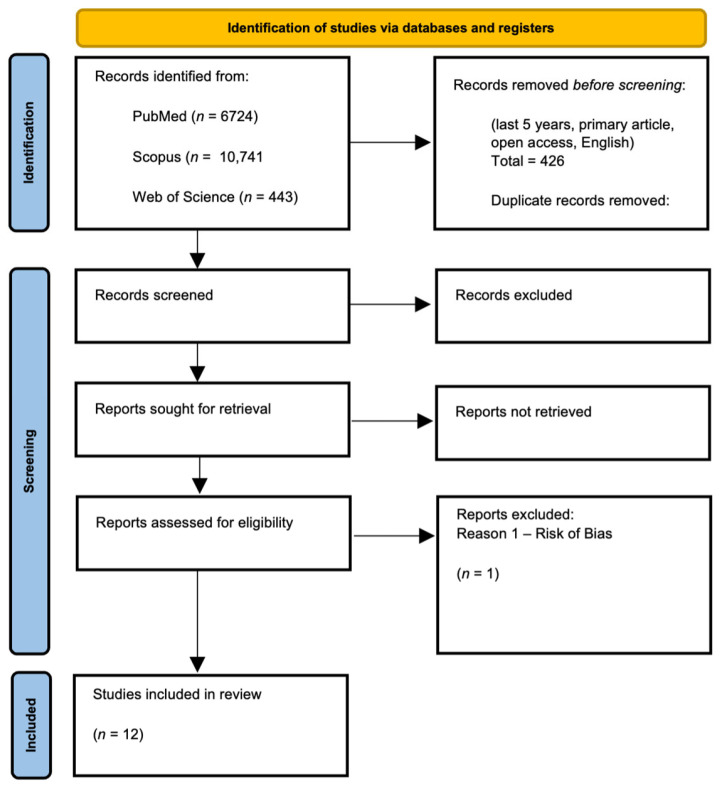
PRISMA Flow Diagram.

A total of 426 records were identified through database searches in PubMed, Scopus, and Web of Science. After removing duplicates, 422 unique records were screened by title and abstract, leaving 29 articles for full-text review. Following full-text assessment and risk of bias evaluation (with details in Appendix B), 13 studies met all inclusion criteria. One study [14] was subsequently excluded due to critical risk of bias, leaving 12 studies for the final synthesis [Appendix B]. The PRISMA flow diagram is presented in Figure 1.

### 3.2. Characteristics of Included Studies

Table 1 summarizes the characteristics of the 12 included studies, which were published between 2020–2025 across diverse settings (e.g., USA, China, Europe). Designs included 5 randomized controlled trials, 4 observational studies, and 3 feasibility or pilot studies. Sample sizes ranged from 35 to 280 participants. Studies included populations with mean or median ages >65 years, populations in which at least 50% of participants were aged ≥65 years, or studies reporting subgroup analyses specifically for individuals aged ≥65 years. All included studies met the predefined age-related eligibility criteria. All included studies focused primarily on CGM methods. No eligible studies on non-invasive glucose monitoring devices specifically targeting older adults were identified (see Table 1).

### 3.3. Risk of Bias Assessment

**Figure 2:**
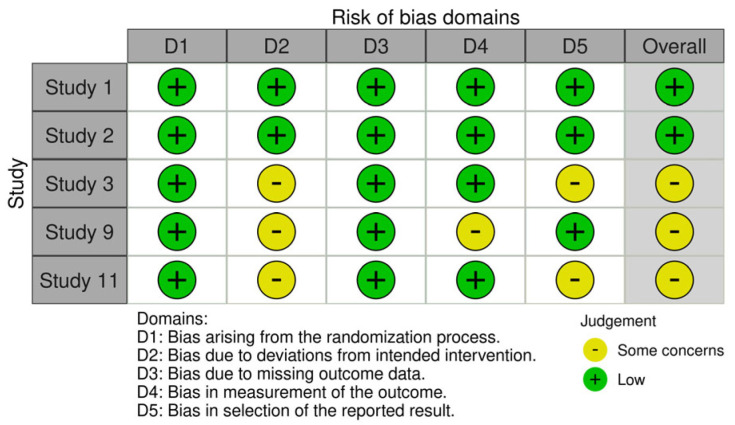
ROB II analysis.

**Figure 3:**
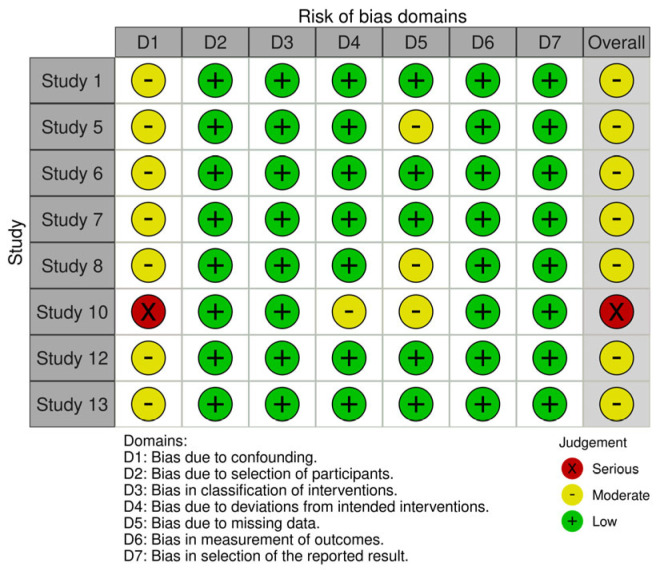
ROBINS I analysis.

Risk of bias assessments for the included studies are detailed in Appendix B. Among the studies undergoing methodological assessment, two were judged to have low risk of bias, ten had moderate risk of bias, and one study [14] was excluded due to critical risk of bias, leaving 12 studies in the final synthesis, as shown in Figure 2 and Figure 3. Common concerns included small sample sizes, lack of consistent blinding, and incomplete outcome data.

### 3.4. Main Findings

Pratley et al. [15] conducted a randomized clinical trial involving 203 older adults (aged ≥60 years) with type 1 diabetes evaluating the effectiveness of continuous glucose monitoring (CGM) versus traditional blood glucose monitoring (BGM) in reducing hypoglycemia. The primary outcome was the percentage of time participants spent with glucose levels below 70 mg/dL. Over a six-month period, CGM users experienced a small but statistically significant reduction in hypoglycemia compared to those using BGM. While the findings support CGM’s potential benefit in this age group, the study also emphasized the need for further investigation into its long-term effects.

In a follow-up to a 26-week randomized clinical trial, Miller et al. [16] assessed glycemic outcomes over 52 weeks in older adults with type 1 diabetes using continuous glucose monitoring (CGM). Participants who continued CGM use (CGM-CGM cohort) maintained significant improvements in hypoglycemia reduction and time in range (TIR), while those who switched from blood glucose monitoring to CGM (BGM-CGM cohort) also demonstrated notable glycemic benefits. These results suggest a potential benefit of sustained CGM use in older adults at risk of hypoglycemia, though the observational nature of the extension phase limits causal inference.

Chu et al. [17] explored the effectiveness of continuous glucose monitoring systems (CGMS) in managing glycemic control among critically ill ICU patients. While CGMS use was associated with an increased Time in Range (TIR), indicating improved glycemic control, it did not significantly affect overall clinical outcomes. Interestingly, the control group spent less time in hypoglycemia, and insulin dosage requirements were similar across both groups. These findings suggest a limited and context-dependent role for CGMS in ICU settings; well-powered prospective trials are needed before it can be considered a viable alternative to point-of-care monitoring (POCM).

Chao et al. [18] examined the role of continuous glucose monitoring (CGM) in optimizing treatment for individuals with insulin-treated type 1 and type 2 diabetes. The study emphasized the benefits of the ‘Urgent Low Soon’ alert feature integrated into the updated G6 algorithm, which led to a notable reduction in severe hypoglycemic episodes and overall improvement in glycemic control compared to traditional self-monitoring of blood glucose. These findings suggest that CGM may support diabetes management and improve patient safety.

Munshi et al. [19] investigated the impact of diabetes-related technologies on glycemic outcomes in older adults (≥65 years) with type 1 diabetes. The study found that users of continuous glucose monitoring (CGM) and insulin pumps achieved better glycemic control, demonstrated by lower A1c levels and reduced risk of hypoglycemia. However, these individuals also reported greater impairment in hypoglycemia awareness and increased diabetes-related distress. The findings underscore the importance of individualized technology selection and the need for larger studies to guide optimal use in this population.

Zelnick et al. [20] assessed the reliability of glycemic biomarkers, HbA1c, glycated albumin, and fructosamine, compared to continuous glucose monitoring (CGM) in patients with type 2 diabetes across different levels of kidney function (eGFR). The study highlighted that HbA1c may be an unreliable indicator of glycemic control in individuals with chronic kidney disease due to variability and potential bias. CGM was identified as a valuable tool for capturing short-term glucose fluctuations and hypoglycemic events.

Toschi et al. [21] examined how continuous glucose monitoring (CGM)-derived metrics relate to HbA1c and hypoglycemia risk in older adults with type 1 diabetes. The study found that participants with a high coefficient of variation (CV) spent significantly more time in hypoglycemia than those with low CV, despite having similar A1c levels. These results highlight the limitations of HbA1c alone and support the use of CGM metrics such as CV and glucose management indicator (GMI) as complementary tools for better identifying hypoglycemia risk. These findings suggest that CGM-derived metrics may complement HbA1c for hypoglycemia risk stratification in older adults. However, prospective validation of these metrics as clinical decision-support tools remains warranted.

Bosi et al. [22] conducted a prospective observational cohort study to evaluate the effectiveness of flash glucose monitoring versus self-monitoring of blood glucose (SMBG) in individuals with type 2 diabetes on basal-bolus insulin therapy. Over a three to six-month period, the flash monitoring group experienced significantly greater reductions in HbA1c compared to the SMBG group. These results highlight the utility of continuous glucose monitoring, especially in patients with obesity, as a means of improving glycemic control in type 2 diabetes management.

Shang et al. [23] conducted a randomized controlled trial to assess the impact of continuous glucose monitoring (CGM) on 28-day in-ICU mortality among 124 frail, critically ill COVID-19 patients. The study demonstrated a significant reduction in mortality (hazard ratio: 0.18) and a decrease in hypoglycemia events in the CGM group compared to those managed with point-of-care glucose testing. These results highlight the potential clinical benefits of CGM in improving outcomes for critically ill patients in ICU settings.

Kahkoska et al. [24], building on findings from the WISDM study, confirmed that continuous glucose monitoring (CGM) was associated with reduced hypoglycemia in older adults with type 1 diabetes compared to traditional blood glucose monitoring (BGM). Using machine learning methods, the study developed a data-driven decision rule to identify individuals who would benefit most from CGM, thereby optimizing time spent in hypoglycemia. These findings emphasize the potential of CGM for personalized treatment strategies, and future research is encouraged to evaluate its utility in optimizing broader glycemic outcomes beyond hypoglycemia.

Krajnc et al. [25] assessed the glycemic control and hypoglycemia risk in type 2 diabetes patients undergoing low-premixed insulin therapy, using real-world data from 35 individuals monitored with the Dexcom G6 system. The study reported an average time in range (TIR) of 62.1% and a low incidence of severe hypoglycemia (time below range, TBR, at 0.8%). While participants met targets for TIR and time above range (TAR), they did not achieve the recommended TBR target for older or high-risk populations. These results support the utility of continuous glucose monitoring (CGM) as a valuable tool for improving glycemic management in this group.

Barua et al. [26] examined the accuracy of continuous glucose monitoring (CGM) versus traditional fingerstick methods in measuring postprandial plasma glucose in nondiabetic older adults. Involving 10 participants over 9 meals, glucose was tracked for 8 h post-consumption. The study found fingerstick measurements to be more accurate, with a mean absolute relative difference (MARD) of 8.0%, compared to 13.7% for CGM. CGM tended to underestimate glucose excursions and time in range, suggesting that caution is warranted when applying CGM for precision nutrition or diabetes risk prediction in non-diabetic older adults, particularly given the very small sample size (*n* = 10).

### 3.5. Synthesis of Findings

To avoid overgeneralization, findings were synthesized by outcome domain rather than as a single global estimate of effect. The main outcome domains were hypoglycemia, glycemic outcomes, including time in range (TIR) and HbA1c, diagnostic accuracy, usability/adherence, and economic implications. Claims for each domain were restricted to studies that directly measured the respective outcome, and numerical effect estimates were reported where available.

### 3.6. Certainty of Evidence

The certainty of evidence for the main patient-important outcomes was assessed using the GRADE approach. Overall, the certainty of evidence was moderate for hypoglycemia, time in range (TIR)/glycemic profile, HbA1c, and usability/adherence, as presented in Table 2. Across these outcomes, the main reason for downgrading was indirectness, reflecting differences in diabetes type, care setting, and outcome definitions among the included studies. Risk of bias was not considered serious for the main outcomes because the randomized evidence was generally judged to be at low risk or to raise only some concerns. The non-randomized studies were mostly at moderate or low risk, and the study with unacceptable risk of bias was excluded from the final synthesis.

Across studies, the most consistent pattern was observed in the reduction of hypoglycemia and improvement in time in range (TIR), particularly in outpatient older adults with insulin-treated diabetes. By contrast, evidence for HbA1c was more variable, and findings from inpatient or ICU-based studies were less directly comparable because of differences in clinical severity, monitoring protocols, and outcome definitions. Usability findings were generally favorable, particularly for newer-generation systems, but were reported heterogeneously and often descriptively. Overall, heterogeneity across diabetes type, care setting, device generation, and reported endpoints limited direct cross-study comparability and supported the decision to use a narrative rather than a quantitative synthesis.

### 3.7. Hypoglycemia and Time in Range Assessment

Hypoglycemia outcomes were analyzed separately from broader glycemic endpoints. Direct evidence was provided by Pratley et al. [15], Miller et al. [16], Chao et al. [18], Munshi et al. [19], Shang et al. [23], Kahkoska et al. [24], and Krajnc et al. [25]. Pratley et al. [15] reported an approximately 27 min/day reduction in hypoglycemia exposure with CGM in older adults with type 1 diabetes, and Miller et al. [16] showed that this benefit was sustained over 12 months. Chao et al. [18] reported fewer severe hypoglycemic episodes, while Shang et al. [23] observed fewer hypoglycemic events in frail, critically ill patients monitored with CGM. Krajnc et al. [25] reported a time below range (TBR) of 0.8% in a real-world cohort of older adults with type 2 diabetes. According to GRADE, the certainty of evidence for hypoglycemia was moderate, indicating that CGM probably reduces hypoglycemia exposure in older adults.

### 3.8. Glycemic Outcomes

Glycemic outcomes were analyzed separately from hypoglycemia and included both time in range (TIR)/glycemic profile and HbA1c. Direct evidence for TIR and related CGM-derived glycemic metrics was provided by Miller et al. [16], Chu et al. [17], Chao et al. [18], Toschi et al. [21], Shang et al. [23], Kahkoska et al. [24], and Krajnc et al. [25]. Chao et al. [18] reported a 13% increase in TIR among participants aged ≥65, whereas Krajnc et al. [25] reported a mean TIR of 62.1% in a real-world older adult cohort. Additional studies reported improvements in glycemic profile metrics such as coefficient of variation and glucose management indicator, although exact estimates were not consistently reported across all studies. According to GRADE, the certainty of evidence for TIR/glycemic profile was moderate, indicating that CGM probably improves glycemic profiling in older adults.

Direct evidence for HbA1c was provided by Pratley et al. [15], Chao et al. [18], Munshi et al. [19], and Bosi et al. [22]. Chao et al. [18] reported a 1.0% reduction in HbA1c, while Bosi et al. [22] found greater improvements in HbA1c with flash glucose monitoring compared with SMBG. Munshi et al. [19] also reported lower HbA1c among older adults using diabetes technology. Although the magnitude of benefit varied across studies, the overall direction of effect favored CGM-based monitoring approaches. According to GRADE, the certainty of evidence for HbA1c was moderate.

### 3.9. Diagnostic Accuracy

Diagnostic accuracy was analyzed separately from clinical effectiveness. Direct evidence was provided by Zelnick et al. [20], Chu et al. [17], Krajnc et al. [25], and Barua et al. [26]. Barua et al. [26] reported that CGM was less accurate than fingerstick measurements for postprandial plasma glucose in non-diabetic older adults, with a mean absolute relative difference (MARD) of 13.7% for CGM compared with 8.0% for fingerstick testing. In contrast, Krajnc et al. [25] reported a MARD of 9.0% for Dexcom G6 in a real-world older adult cohort. Zelnick et al. [20] showed that CGM may better reflect short-term glycemic variability than HbA1c in patients with chronic kidney disease, whereas Chu et al. [17] suggested that CGM may offer practical advantages over intermittent point-of-care monitoring in ICU settings. Taken together, these studies indicate that findings on diagnostic performance are mixed and should be interpreted separately from claims regarding hypoglycemia reduction or glycemic improvement.

### 3.10. The Accuracy of Continuous Glucose Monitoring (CGM)

The accuracy of continuous glucose monitoring (CGM) devices is an important factor in optimizing diabetes care, particularly in older or high-risk populations. Several studies suggest that CGM may offer advantages over traditional methods such as blood glucose monitoring (BGM) and point-of-care monitoring (POCM), although findings are not entirely consistent.

Pratley et al. [15] reported a reduction in hypoglycemia time with CGM, while Miller et al. [16] noted that earlier-generation systems (G5) required calibration, highlighting the improved performance of newer-generation sensors such as the G6. Chu et al. [17] observed that real-time CGM measurements were more reliable than POCM in ICU settings. Chao et al. [18] reported that sensor placement may influence measurement accuracy, and Munshi et al. [19] found improved glycemic control and reduced excursions among CGM users.

Zelnick et al. [20] suggested that CGM may better capture short-term glycemic variability than HbA1c, particularly in patients with chronic kidney disease. Similarly, Toschi et al. [21] emphasized that CGM-derived metrics, such as coefficient of variation and glucose management indicator, may provide additional value beyond HbA1c alone for assessing hypoglycemia risk. Bosi et al. [22] reported that CGM detected a greater proportion of glycemic excursions compared with SMBG, including events that might otherwise be missed.

In critically ill populations, Shang et al. [23] highlighted potential advantages of CGM over SMBG in ICU care, although limitations remain in intermittently scanned systems. Kahkoska et al. [24] further supported the role of CGM in improving hypoglycemia detection. Krajnc et al. [25] reported a mean absolute relative difference (MARD) of 9.0% for the Dexcom G6, indicating acceptable accuracy in a real-world older adult cohort. Conversely, Barua et al. [26] found that fingerstick measurements were slightly more accurate than CGM for postprandial glucose assessment in non-diabetic older adults.

Overall, these findings suggest that while CGM provides clinically useful and often acceptable accuracy, its performance may vary depending on the population, clinical setting, and device generation.

### 3.11. Usability and Adherence to Continuous Glucose Monitoring (CGM)

The usability of continuous glucose monitoring (CGM) systems and adherence to these systems are important determinants of their effectiveness, particularly among older adults and medically complex populations. Pratley et al. [15] reported high adherence among older adults, with 83% using CGM for at least six days per week, supporting the feasibility of CGM in this population. Miller et al. [16] reported even higher adherence (median >90%) with the Dexcom G6 system, which the authors attributed to its calibration-free design and automated insertion, although smartphone compatibility issues occasionally limited app use. In contrast, Chu et al. [17] highlighted practical challenges associated with CGM systems requiring frequent calibrations in critically ill patients. Chao et al. [18] suggested that structured CGM implementation through phased protocols may support adherence. Munshi et al. [19] acknowledged the presence of cognitive and physical barriers in older adults, although participants using CGM or insulin pumps showed more favorable glycemic outcomes. Bosi et al. [22] emphasized the potential contribution of policy and structural support to sustained device use. Krajnc et al. [25] supported the real-world feasibility of CGM in older adults, particularly in higher-risk settings, while Kahkoska et al. [24] noted that adherence may also be influenced by age-related differences in technological comfort and treatment preferences. Overall, the available evidence suggests that CGM is generally feasible and acceptable in older adults, particularly with newer-generation systems, although usability outcomes were reported heterogeneously across studies.

### 3.12. Clinical Effectiveness of CGM

The clinical efficacy of continuous glucose monitoring (CGM) is supported across various populations and care settings, with evidence suggesting improvements in glycemic control and reductions in hypoglycemia. Pratley et al. [15] and Miller et al. [16] reported significant reductions in hypoglycemia and HbA1c levels among older adults using CGM, suggesting meaningful clinical benefits. Similarly, Chao et al. [18] found a 1.0% HbA1c reduction and a 13% increase in time in range (TIR) in participants aged ≥65. Chu et al. [17] observed enhanced glycemic control and TIR in ICU patients using CGM, while Shang et al. [23] reported lower ICU mortality and hypoglycemia rates in CGM users. Participants using diabetes-related technologies in Munshi et al. [19] experienced better glycemic profiles and lower hypoglycemia risk. Bosi et al. [22] similarly reported improvements in HbA1c with flash glucose monitoring. Studies by Toschi et al. [21] and Kahkoska et al. [24] emphasized CGM-derived metrics such as coefficient of variation (CV) and glucose management indicator (GMI) as valuable for guiding clinical management. Zelnick et al. [20] highlighted limitations of HbA1c and the complementary role of CGM in patients with chronic kidney disease. Krajnc et al. [25] noted underachievement in time below range (TBR) targets, despite adequate TIR.

### 3.13. Economic Considerations

While direct cost-effectiveness analyses are largely absent across the included studies, several studies suggest potential health economic benefits associated with continuous glucose monitoring (CGM) use. Pratley et al. [15] noted that reductions in hypoglycemic events may lead to fewer emergency interventions and hospitalizations, suggesting long-term cost savings despite the upfront costs of CGM devices. Miller et al. [16] and Chu et al. [17] echoed this perspective, with improved clinical outcomes suggesting downstream economic advantages through reduced complications. Finally, Bosi et al. [22] mentioned reimbursement of flash glucose monitoring systems (FGMS) by national health authorities, suggesting potential policy-level cost-effectiveness. Therefore, economic implications should be interpreted as suggestive rather than conclusive.

### 3.14. Challenges in Geriatric Population

Age-related physiological changes, comorbidities, and disease-specific factors present substantial challenges to effective diabetes management, particularly in older adults and critically ill patients. Pratley et al. [15] emphasized that baseline hypoglycemia and increased glycemic variability in older adults necessitate more precise monitoring strategies, such as CGM. Miller et al. [16] noted the risk of hypoglycemia and impaired awareness in older adults with type 1 diabetes (T1D), reinforcing the need for CGM in this context. Similarly, Munshi et al. [19] highlighted that aging-related cognitive decline and comorbidities require cautious implementation of technology-based solutions.

### 3.15. Physical and Cognitive Barriers in Geriatric Population

In older adults, impaired hand dexterity and reduced bimanual coordination present significant barriers to effective diabetes self-management, particularly when using glucose monitoring technologies. Although not directly assessed, Pratley et al. [15] acknowledged these challenges as important considerations for CGM implementation in older adult populations. Devices that minimize manual manipulation, such as those with automated insertion, may offer enhanced usability. Supporting this, Miller et al. [16] highlighted the Dexcom G6 sensor’s automatic insertion feature, which could alleviate challenges associated with manual dexterity. Barua et al. [26] did not assess dexterity directly but implied that CGM systems requiring less manual dexterity than fingerstick meters may be preferable for users with physical limitations.

Cognitive decline and frailty are prevalent challenges in older adults that significantly affect the management of type 1 diabetes. Pratley et al. [15] found that CGM use provided consistent glycemic benefits across varying levels of cognitive function, suggesting potential applicability across a range of cognitive stress. However, the ability to interpret glucose trends and make real-time adjustments may be compromised in cognitively impaired individuals, underscoring the importance of targeted education and supportive care. Shang et al. [23] implied that CGM might help reduce the burden of frequent manual glucose checks in frail or cognitively impaired patients, thereby potentially enhancing feasibility and adherence in this vulnerable group.

## 4. Discussion

This systematic review suggests that CGM may offer benefits for older adults, particularly by reducing hypoglycemia exposure and improving glycemic profiling. However, the evidence base is heterogeneous with respect to diabetes type, care setting, study design, device generation, comparators, and outcome definitions, and the certainty of evidence for the main outcomes was moderate rather than high. Additionally, several included studies were subject to moderate risk of bias, which should be considered when interpreting the overall strength of the evidence. The most consistent benefits were observed in outpatient older adults with insulin-treated diabetes, whereas findings from inpatient and ICU settings were less directly comparable.

The performance of newer CGM systems, including Dexcom G6 and flash monitoring platforms, supports their increasing adoption in clinical practice. Although occasional underestimation of glucose values has been reported [26], CGM often provides more clinically informative glycemic data than traditional self-monitoring of blood glucose (SMBG), especially in detecting glycemic variability and hypoglycemia [21].

Importantly, although this review was designed to evaluate both continuous and non-invasive glucose monitoring technologies, no eligible studies investigating fully non-invasive approaches in older adults were identified. This finding reflects a genuine gap in the current literature rather than a limitation of the search methodology, as the search strategy explicitly included a broad range of non-invasive technologies and related terminology. The absence of such studies suggests that non-invasive glucose monitoring remains insufficiently validated in older adults, highlighting an important direction for future research.

Device usability remains a critical factor, especially in older adults with cognitive impairment, frailty, or reduced manual dexterity. Features such as automated sensor insertion and factory calibration appear to improve feasibility and support adherence in these populations [15,16].

Beyond clinical performance metrics, patient experience and acceptability are also important when evaluating CGM, particularly in complex clinical settings. Qualitative research may provide insights that quantitative studies do not fully capture, including perceived usability, motivational factors, and barriers to sustained adherence. For example, Cuevas-Budhart et al. explored the experiences of patients with type 2 diabetes using CGM during dialysis sessions and highlighted patient-reported perspectives on device usability, perceived benefits, and adherence in a medically complex population [27]. Although such evidence remains limited and was not a major focus of the geriatric literature identified in this review, it underscores the value of incorporating patient-centered and qualitative approaches in future studies of CGM use in older adults.

Despite these encouraging findings, several limitations should be acknowledged. Many studies did not include formal cost-effectiveness analyses, limiting conclusions regarding the economic impact of CGM adoption. Furthermore, the applicability of findings from some trials is limited by small sample sizes, short follow-up durations, and the exclusion of individuals with severe cognitive or physical disabilities. In acute care settings, variability in device performance and calibration protocols may also affect outcome reliability. Additionally, heterogeneity in study design, population characteristics, and outcome reporting limits direct comparability across studies and should be considered when interpreting these findings, as these factors reduce the certainty and generalizability of the conclusions.

Overall, the most consistent pattern favored CGM in reducing hypoglycemia and improving glycemic profiling, particularly in outpatient older adults with insulin-treated diabetes. Evidence regarding HbA1c, diagnostic accuracy, and economic implications was more variable, and findings from inpatient or ICU settings were less directly comparable. Future research should focus on long-term outcomes, personalized strategies for individuals with cognitive or physical impairments, and comprehensive economic evaluations to inform clinical guidelines and health policy.

Notably, this review also revealed a lack of primary research on non-invasive glucose monitoring in older adults. Although the initial objective included comparison with non-invasive approaches, only studies evaluating CGM met the inclusion criteria. This highlights a substantial gap in the literature and underscores the need for targeted investigations into the feasibility, accuracy, and usability of non-invasive glucose monitoring technologies in older adults. The interpretation of current findings should therefore remain cautious until higher-quality evidence becomes available. Particular attention should be given to age-related skin, behavioral, and metabolic changes that may influence device performance and the sustained use of these technologies.

## 5. Conclusions

This systematic review suggests that continuous glucose monitoring (CGM) may improve glycemic control and reduce hypoglycemia in older adults across a range of clinical settings. Advances in device design may also support adherence in individuals with cognitive impairment, frailty, or reduced manual dexterity. Nevertheless, the successful integration of CGM into geriatric diabetes care requires education tailored to the needs of older adults, along with ongoing support to address usability challenges and optimize clinical benefit.

A major limitation of this review is the absence of eligible primary studies on fully non-invasive glucose monitoring technologies in older adults, precluding any conclusions regarding these approaches in this population. More broadly, the available evidence for CGM remains heterogeneous and includes studies with variable design, setting, and methodological quality. Further well-designed studies with longer follow-up, clearer reporting of patient-centered outcomes, and better representation of older adults with cognitive or physical impairments are needed to strengthen the evidence base.

## Figures and Tables

**Table 1:** Characteristics of Included Studies.

No.	Author	Country	Study Design	Sample Size	Population (Age)	Diabetes Type	Setting	Device/ Platform	Follow-Up Duration	Key Numeric Findings	Intervention/Comparator	Outcome	Reference
1	Pratley et al., 2020	USA	RCT	203	70	T1D	Outpatient	CGM (Dexcom)	6 months	↓ hypoglycemia time (~27 min/day reduction)	CGM vs. SBGM	HbA1c, glycemia	[15]
2	Miller et al., 2022	USA	RCT	198	+/−65	T1D	Outpatient	Dexcom G5/G6	12 months	sustained ↓ hypoglycemia, ↑ TIR	CGM vs. SBGM	HbA1c, glycemia	[16]
3	Chu et al., 2024	China	RCT	96	70	Mixed/ICU	Inpatient (ICU)	CGM	ICU stay	↑ TIR, no mortality difference	CGM vs. SBGM	HbA1c, TIR	[17]
4	Chao et al., 2023	USA	Interventional	63	Separately for 65	T1D + T2D	Outpatient	Dexcom G6	weeks–months	↓ severe hypoglycemia, ↑ glycemic control	CGM vs. SBGM	HbA1c, TIR, hypoglycemia	[18]
5	Munshi et al., 2022	USA	Observational	165	70	T1D	Outpatient	CGM + insulin pump	cross-sectional	↓ HbA1c, ↑ hypoglycemia awareness issues	CGM vs. SBGM	HbA1c, glycemia	[19]
6	Zelnick et al., 2020	USA	Observational	104	68	T2D	Outpatient	CGM	cross-sectional	CGM better than HbA1c in CKD patients	CGM	HbA1c, fructosamine, glycated albumin	[20]
7	Toschi et al., 2020	USA	Cohort	130	71	T1D	Outpatient	CGM	observational	high CV → ↑ hypoglycemia risk	CGM vs. HbA1c	HbA1c, CV, GMI	[21]
8	Bosi et al., 2022	Italy	Cohort	322	67.2	T2D	Outpatient	Flash CGM	3–6 months	↓ HbA1c vs. SMBG	CGM vs. SBGM	HbA1c	[22]
9	Shang et al., 2025	China	RCT	80	78.3	T2D/ICU	Inpatient	CGM	28 days	↓ mortality (HR ~0.18), ↓ hypoglycemia	CGM vs. SBGM	TIR, Glycemia variation	[23]
10	Kahkoska et al., 2024	USA	RCT	203	67.9	T1D	Outpatient	CGM	secondary analysis	↓ hypoglycemia, ML prediction benefit	CGM vs. SBGM	TIR, CV	[24]
11	Krajnc et al., 2023	Slovenia	Observational	35	70.4	T2D	Real-world outpatient	Dexcom G6	observational	TIR ~62%, low severe hypoglycemia	CGM	HbA1c, TIR	[25]
12	Barua et al., 2022	USA	Observational	10	68.6	Non-diabetic older adults	Outpatient	CGM	acute meal testing	CGM less accurate than fingerstick (MARD ~13.7%)	CGM vs. plasma glucose	Glucose variation	[26]

In the table, arrows indicate direction of change or association: ↑ increase, ↓ decrease, → leads to/is associated with.

**Table 2:** GRADE Evidence Profile for the Main Outcomes.

Outcome	Studies Directly Contributing	Study Design Informing the Body of Evidence	Risk of Bias	Inconsistency	Indirectness	Imprecision	Publication Bias	Overall Certainty (GRADE)	Outcome-Specific Conclusion
Hypoglycemia	Pratley et al. [15], Miller et al. [16], Chao et al. [18], Munshi et al. [19], Shang et al. [23], Kahkoska et al. [24], Krajnc et al. [25]	Mainly randomized evidence, supported by cohort/observational studies	not serious	not serious	serious	not serious	undetected	Moderate	CGM probably reduces hypoglycemia exposure in older adults. In the current manuscript, numerical findings include an approximately 27 min/day reduction in hypoglycemia time in Pratley et al. [15] and a TBR of 0.8% in Krajnc et al. [25].
Time in range (TIR)/glycemic profile	Miller et al. [16], Chu et al. [17], Chao et al. [18], Toschi et al. [21], Shang et al. [23], Kahkoska et al. [24], Krajnc et al. [25]	Mainly randomized evidence, supported by non-randomized studies	not serious	not serious	serious	not serious	undetected	Moderate	CGM probably improves TIR and glycemic profiling in older adults. In the current manuscript, numerical findings include a 13% increase in TIR in Chao et al. [18] and a mean TIR of 62.1% in Krajnc et al. [25].
HbA1c	Pratley et al. [15], Chao et al. [18], Munshi et al. [19], Bosi et al. [22]	Randomized and comparative non-randomized evidence	not serious	serious	not serious	not serious	undetected	Moderate	CGM probably improves HbA1c in older adults, although the magnitude of benefit varies across studies. In the current manuscript, Chao et al. [18] reported a 1.0% HbA1c reduction.
Usability/adherence/acceptability	Pratley et al. [15], Miller et al. [16], Chu et al. [17], Chao et al. [18], Munshi et al. [19], Bosi et al. [22], Kahkoska et al. [24], Krajnc et al. [25]	Randomized and non-randomized evidence with direct reporting of feasibility/adherence	not serious	not serious	serious	not serious	undetected	Moderate	CGM probably has acceptable usability and adherence in older adults, especially with newer-generation systems. In the current manuscript, Pratley et al. [15] reported 83% use for at least 6 days/week and Miller et al. [16] reported median adherence >90%.

a. GRADE was applied only to the main patient-important outcomes most directly aligned with the review objective. b. Risk of bias was not downgraded because the randomized evidence was judged as low risk or with some concerns; the non-randomized studies were mostly at moderate risk, and the study with unacceptable bias was excluded from synthesis. c. Indirectness was downgraded by one level because the included studies covered heterogeneous populations and settings, including type 1 diabetes, type 2 diabetes, outpatient care, and frail or critically ill inpatient populations. d. Publication bias could not be formally tested because no meta-analysis was performed and the number of studies per outcome was limited.

## Data Availability

The data supporting the findings of this study are derived from publicly available sources. Extracted data are available from the corresponding author upon reasonable request.

## Supplementary Materials

The following supporting information can be downloaded at: https://www.mdpi.com/article/10.3390/jcm15093194/s1, Table S1: PRISMA 2020 Checklist.

## Abbreviations

The following abbreviations are used in this manuscript:

CGM	Continuous Glucose Monitoring
FGMS	Flash Glucose Monitoring Systems
HbA1c	Glycated Hemoglobin
BGM	Blood Glucose Monitoring
SBGM	Self Blood Glucose Monitoring
SMBG	Self Monitoring of Blood Glucose
NIGM	Non-Invasive Glucose Monitoring
T2DM	Type 2 Diabetes Mellitus
DM	Diabetes Mellitus
TIR	Time in Range
TAR	Time Above Range
MARD	Mean Absolute Relative Difference
CV	Coefficient of Variation
DMSM	Diabetes Self-Management
GMI	Glucose Management Indicator
ICU	Intensive Care Unit
POCM	Point of Care Monitoring

## Appendix A

The comprehensive literature search was conducted in PubMed, Scopus, and Web of Science. The initial search was performed on 15 May 2025 and updated on 20 January 2026. The final search date was therefore 20 January 2026. The search was designed to identify primary studies published between January 2020 and December 2025 that investigated non-invasive and continuous glucose monitoring methods in geriatric populations. Each search combined controlled vocabulary terms (e.g., MeSH) and free-text keywords relating to population, intervention, and outcomes of interest. No filters were applied other than publication date and English language. The detailed search strategies used for each database are listed below.

The PubMed search strategy was as follows: (“age” OR “aged” OR “geriatric*” OR “geriatr*” OR “older adult*” OR “elder*” OR “old” OR “aging” OR “older” OR “old adult*” OR “high age” OR “advanced age” OR “gerontol*” OR “GeriatricAssessment/classification”[Mesh] OR “Aged”[Mesh] OR “Geriatrics/standards”[Mesh] OR “Geriatrics/classification”[Mesh] OR “Geriatrics/methods”[Mesh] OR “Geriatrics/statistics and numerical data”[Mesh]) AND (“blood glucose self monitor*” OR “noninvasive glucose monitor*” OR “non invasive glucose monitor*” OR “non-invasive glucose monitor*” OR “non-invasive blood glucose” OR “noninvasive blood glucose” OR “non invasive blood glucose” OR “wearable glucose monitor*” OR “continuous glucose monitor*” OR “CGM” OR “sensor based glucose monitor*” OR “GlucoWatch” OR “Abbott Freestyle Libre” OR “Dexcom” OR “Continuous Glucose Monitoring Device” OR “CGM device*” OR “Implantable Glucose Monitoring Devices” OR “spectroscopy” OR “polarimetry” OR “ultrasound” OR “glycemia” OR “Continuous Glucose Monitoring/adverse effects”[Mesh] OR “Continuous Glucose Monitoring/classification”[Mesh] OR “Continuous Glucose Monitoring/economics”[Mesh] OR “Continuous Glucose Monitoring/ethics”[Mesh] OR “Continuous Glucose Monitoring/history”[Mesh] OR “Continuous Glucose Monitoring/instrumentation”[Mesh] OR “Continuous Glucose Monitoring/methods”[Mesh] OR “Continuous Glucose Monitoring/mortality”[Mesh] OR “Continuous Glucose Monitoring/nursing”[Mesh] OR “Continuous Glucose Monitoring/psychology”[Mesh] OR “Continuous Glucose Monitoring/standards”[Mesh] OR “Spectrum Analysis, Raman/history”[Mesh] OR “Spectrum Analysis, Raman/instrumentation”[Mesh] OR “Spectrum Analysis, Raman/methods”[Mesh] OR “Spectrum Analysis, Raman/standards”[Mesh]) AND (“capillary blood glucose” OR “fingerstick” OR “invasive glucose monitor*” OR “glucose test*” OR “invasive glucose test*” OR “SBGM” OR “self blood glucose monitoring” OR “finger prick” OR “self-monitoring of blood glucose” OR “SMBG” OR “SMBG” OR “Blood Sugar Self-Monitoring” OR “Blood Sugar Self Monitoring” OR “self-monitoring” OR “ Self monitoring” OR “Home Blood Glucose Monitoring” OR “Blood Glucose Self-Monitoring/adverse effects”[Mesh] OR “Blood Glucose Self-Monitoring/classification”[Mesh] OR “Blood Glucose Self-Monitoring/economics”[Mesh] OR “Blood Glucose Self-Monitoring/history”[Mesh] OR “Blood Glucose Self-Monitoring/instrumentation”[Mesh] OR “Blood Glucose Self-Monitoring/methods”[Mesh] OR “Blood Glucose Self-Monitoring/mortality”[Mesh] OR “Blood Glucose Self-Monitoring/nursing”[Mesh] OR “Blood Glucose Self-Monitoring/psychology”[Mesh] OR “Blood Glucose Self-Monitoring/standards”[Mesh] OR “Blood Glucose Self-Monitoring/statistics and numerical data”[Mesh] OR “Glycemic Control/economics”[Mesh] OR “Glycemic Control/ethics”[Mesh] OR “Glycemic Control/history”[Mesh] OR “Glycemic Control/instrumentation”[Mesh] OR “Glycemic Control/methods”[Mesh] OR “Glycemic Control/mortality”[Mesh] OR “Glycemic Control/nursing”[Mesh] OR “Glycemic Control/psychology”[Mesh] OR “Glycemic Control/standards”[Mesh] OR “Glycemic Control/statistics and numerical data”[Mesh] OR “Glycemic Control/trends”[Mesh] OR “Blood Glucose/analysis”[Mesh] OR “Blood Glucose/chemistry”[Mesh] OR “Blood Glucose/physiology”[Mesh]) AND (“treatment adherence and compliance” OR “patient satisfaction” OR “quality of life” OR “patient comfort” OR “glycemic control” OR “glycemia control” OR “HbA1c” OR “glycated hemoglobin” OR “hypoglycemia” OR “hyperglycemia” OR “life style*” OR “Glycated Hemoglobin/administration and dosage”[Mesh] OR “Glycated Hemoglobin/adverse effects”[Mesh] OR “Glycated Hemoglobin/analysis”[Mesh] OR “Glycated Hemoglobin/chemistry”[Mesh] OR “Glycated Hemoglobin/classification”[Mesh] OR “Glycated Hemoglobin/drug effects”[Mesh] OR “Glycated Hemoglobin/genetics”[Mesh] OR “Glycated Hemoglobin/history”[Mesh] OR “Glycated Hemoglobin/immunology”[Mesh] OR “Glycated Hemoglobin/physiology”[Mesh] OR “Glycated Hemoglobin/standards”[Mesh] OR “Glycated Hemoglobin/supply and distribution”[Mesh] OR “Glycated Hemoglobin/therapeutic use”[Mesh] OR “Glycated Hemoglobin/toxicity”[Mesh] OR “Patient Satisfaction/economics”[Mesh] OR “Patient Satisfaction/ethnology”[Mesh] OR “Patient Satisfaction/legislation and jurisprudence”[Mesh] OR “Patient Satisfaction/statistics and numerical data”[Mesh] OR “Quality of Life/legislation and jurisprudence”[Mesh] OR “Quality of Life/psychology”[Mesh] OR “Life Style/ethnology”[Mesh] OR “Life Style/history”[Mesh] OR “Healthy Lifestyle/classification”[Mesh] OR “Healthy Lifestyle/drug effects”[Mesh] OR “Healthy Lifestyle/ethics”[Mesh] OR “Healthy Lifestyle/physiology”[Mesh]).

The search we performed on Scopus was the next one: (“age” OR “aged” OR “geriatric*” OR “geriatr*” OR “older adult*” OR “elder*” OR “old” OR “aging” OR “older” OR “old adult*” OR “high age” OR “advanced age” OR “gerontol*”) AND (“blood glucose self monitor*” OR “noninvasive glucose monitor*” OR “non invasive glucose monitor*” OR “non-invasive glucose monitor*” OR “non-invasive blood glucose” OR “noninvasive blood glucose” OR “non invasive blood glucose” OR “wearable glucose monitor*” OR “continuous glucose monitor*” OR “CGM” OR “sensor based glucose monitor*” OR “GlucoWatch” OR “Abbott Freestyle Libre” OR “Dexcom” OR “Continuous Glucose Monitoring Device” OR “CGM device*” OR “Implantable Glucose Monitoring Devices” OR “spectroscopy” OR “polarimetry” OR “ultrasound” OR “glycemia”) AND (“capillary blood glucose” OR “fingerstick” OR “invasive glucose monitor*” OR “glucose test*” OR “invasive glucose test*” OR “SBGM” OR “self blood glucose monitoring” OR “finger prick” OR “self-monitoring of blood glucose” OR “SMBG” OR “SMBG” OR “Blood Sugar Self-Monitoring” OR “Blood Sugar Self Monitoring” OR “self-monitoring” OR “ Self monitoring” OR “Home Blood Glucose Monitoring”) AND (“treatment adherence and compliance” OR “patient satisfaction” OR “quality of life” OR “patient comfort” OR “glycemic control” OR “glycemia control” OR “HbA1c” OR “glycated hemoglobin” OR “hypoglycemia” OR “hyperglycemia” OR “life style*”).

The search we performed on Web of Science was the next one: (“age” OR “aged” OR “geriatric*” OR “geriatr*” OR “older adult*” OR “elder*” OR “old” OR “aging” OR “older” OR “old adult*” OR “high age” OR “advanced age” OR “gerontol*”) AND (“blood glucose self monitor*” OR “noninvasive glucose monitor*” OR “non invasive glucose monitor*” OR “non-invasive glucose monitor*” OR “non-invasive blood glucose” OR “noninvasive blood glucose” OR “non invasive blood glucose” OR “wearable glucose monitor*” OR “continuous glucose monitor*” OR “CGM” OR “sensor based glucose monitor*” OR “GlucoWatch” OR “Abbott Freestyle Libre” OR “Dexcom” OR “Continuous Glucose Monitoring Device” OR “CGM device*” OR “Implantable Glucose Monitoring Devices” OR “spectroscopy” OR “polarimetry” OR “ultrasound” OR “glycemia”) AND (“capillary blood glucose” OR “fingerstick” OR “invasive glucose monitor*” OR “glucose test*” OR “invasive glucose test*” OR “SBGM” OR “self blood glucose monitoring” OR “finger prick” OR “self-monitoring of blood glucose” OR “SMBG” OR “SMBG” OR “Blood Sugar Self-Monitoring” OR “Blood Sugar Self Monitoring” OR “self-monitoring” OR “ Self monitoring” OR “Home Blood Glucose Monitoring”) AND (“treatment adherence and compliance” OR “patient satisfaction” OR “quality of life” OR “patient comfort” OR “glycemic control” OR “glycemia control” OR “HbA1c” OR “glycated hemoglobin” OR “hypoglycemia” OR “hyperglycemia” OR “life style*”).

The search covered studies published between January 2020 and December 2025. We used a combination of Medical Subject Headings (MeSH) and free-text terms relevant to the research topic.

The primary search without applying any criteria on PubMed generated 6724 results. After applying the filter regarding the publication date of all the articles, PubMed generated 2704 results. Furthermore, we applied the filter regarding the inclusion of primary studies (Clinical Study, Clinical Trial, Randomized Controlled Trial, Observational Study, Comparative Study, Evaluation Study, Multicenter Study, Validation Study), generating 1095 results. Afterwards, we applied the English language filter, generating 1091 results. After applying the “humans” and age (“>65”) filters, we restricted the search to 441 results. The final filter we applied is the open access source, which generated 282 results.

Secondly, the primary search, by keywords, as described, without applying any criteria on Scopus generated 10,741 results. Filtering by publication date, Scopus generated 4899 results. Applying the filter regarding the area of interest, Medicine, 4170 documents were found. Checking English as language, our search was limited to 3997 results. By applying the open access filter, our search was limited to 2614 results. As a document type, we checked Article type, so we obtained 2124 results. As a publication stage, by choosing “Final”, the search was restricted to 2107 results. As a source type, we checked Journal type, by obtaining 2106 results. Finally, in the Keyword column offered by Scopus, we limited the search to Aged, Blood Glucose Monitoring, Blood Glucose Self-Monitoring, Clinical Trial, Cohort Analysis, Comparative Study, Cross-Sectional Study, Cross-Sectional Studies, Randomized Controlled Trial, Observational Study, Aged, 80 And Over, Continuous Glucose Monitoring, Dexcom G6, Dexcom, Devices, Flash Glucose Monitoring, Freestyle Libre, Glycated Hemoglobin A, HbA1c, Hemoglobin A1c, Glycosylated Hemoglobin, Quality of Life, Self Monitoring, Self Care, Wearable Electronic Devices, excluding Antidiabetic Agent, Middle Aged, Body Mass, Child, Metabolism, Young Adult, Disease Duration, Complication, Adult, Adolescent, Biomarkers, C Peptide, COVID-19, Carbohydrate Intake, Child, Preschool, Drug Effect, Drug Efficacy, Drug Safety, Drug Therapy, Epidemiology, Estimated Glomerular Filtration Rate, Glucagon Like Peptide 1 Receptor Agonist, Pregnancy, Pregnancy Outcome, Pregnant Woman, Preschool Child, School Child, Sulfonylurea, Systolic Blood Pressure, obtaining 110 documents. One article has been shown as retracted, remaining with 109 articles.

Furthermore, doing the same primary search on Web of Science, by keywords, we obtained 443 documents. By restricting to open access publications only, applying the filter regarding the publication date, by limiting the search to English language, by excluding Pediatrics and Veterinary from the fields of interest, focusing on General Medicine, Endocrines and Gerontology, by excluding all the review type articles, we concluded with a total of 35 documents.

The initial search across PubMed, Scopus, and Web of Science yielded a total of 426 records. After removing duplicates, 422 unique articles remained. These were screened by title and abstract, leading to the exclusion of 393 records. The main reasons for exclusion at this stage included studies focusing on unrelated topics, non-primary research articles (e.g., reviews, editorials), populations outside the geriatric age range, irrelevant interventions, or outcomes not aligned with the objectives of this review. As a result, 29 articles were retained for full-text evaluation.

The remaining 29 articles underwent full-text assessment. After applying the same eligibility criteria at this stage, population characteristics, intervention type, and relevance of outcomes, 16 studies were excluded. Thus, 13 articles were left for risk of bias assessment and potential inclusion in the synthesis.

After full-text screening and risk of bias assessment, 12 studies were included in the final synthesis. Of the 13 articles that initially met all inclusion criteria, one was excluded due to a serious risk of bias identified using the ROBINS-I tool, as explained in Appendix B. The search strategy was designed to capture studies on both continuous glucose monitoring (CGM) and non-invasive glucose monitoring methods in the geriatric population. Despite a comprehensive search across PubMed, Scopus, and Web of Science, the majority of included studies focused on CGM. This highlights a current gap in the published literature regarding non-invasive glucose monitoring techniques in older adults.

## Appendix B

All studies that met the inclusion criteria were subjected to a structured risk of bias assessment to evaluate the internal validity of the evidence. For randomized controlled trials (RCTs), we applied the Cochrane Risk of Bias 2 (RoB 2) tool (Figure 2 in the main text). For observational and non-randomized intervention studies, we used the Risk Of Bias In Non-randomized Studies of Interventions (ROBINS-I) tool (Figure 3 in the main text)). Each study was independently assessed by two reviewers across key domains relevant to its design. Disagreements were resolved through discussion or, when necessary, consultation with a third reviewer.

The overall risk of bias for each study was classified as low, moderate, serious, or critical based on tool-specific criteria. One study was excluded from the final synthesis due to being judged at critical risk of bias. The table below (Table A1) summarizes the risk of bias ratings and the justifications for each judgment, as shown in Figure 2 and Figure 3 in the main text.

One study was excluded following ROBINS-I assessment due to critical risk of bias. Although the study design and execution were methodologically sound for a pilot trial, the small sample size, significant dropout imbalance between groups, and logistical challenges affecting intervention adherence elevated the overall risk of bias to a critical level. While the findings offer useful insights for future research design, they were deemed insufficiently robust to inform clinical conclusions within this review.

**Table A1:** Summary of Studies Included in the Review.

No	Author (Year)	Study Design	Risk of Bias Tool Used	Overall Risk of Bias	Justification
1	Pratley et al., 2020	RCT	ROB2	Low	Rigorously conducted and reported
2	Miller et al., 2022	RCT	ROB2	Low	Rigorously conducted and reported
3	Chu et al., 2024	RCT	ROB2	Some concerns	While the trial was well-designed and reported clearly, issues with non-intention-to-treat analysis and selective reporting raise some concerns. The findings suggest CGMS may improve glycemic control metrics in the ICU without significantly affecting mortality, but results should be interpreted with caution.
4	Chao et al., 2023	Single arm, interventional	ROBINS I	Moderate	While the study design and execution are strong for an interventional non-randomized trial, the lack of a parallel comparator group remains the main limitation. The robustness of outcome measurement and adherence to pre-specified analysis plans strengthen its internal validity, but residual confounding and the Hawthorne effect cannot be excluded.
5	Munshi et al., 2022	Cross-sectional	ROBINS I	Moderate	The primary source of concern lies in residual confounding and some incomplete data across subgroups. Despite these, the study design and transparent reporting strengthen its reliability within the limits of observational data.
6	Zelnick et al., 2020	Cross-sectional	ROBINS I	Moderate	Although most domains are at low risk, the primary limitation remains the potential for residual confounding due to the observational nature of the study and complex interactions in CKD-related glycemic variability. The careful study design and transparent reporting mitigate most concerns.
7	Toschi et. al, 2020	Cohort	ROBINS I	Moderate	Although most domains are low risk, moderate risk of bias due to confounding—inherent in the observational design—yields a Moderate overall risk according to the ROBINS-I framework.
8	Bosi et al., 2022	Cohort	ROBINS I	Moderate	Despite strong design features and minimal bias across most domains, the moderate risk of confounding—typical of non-randomized studies—warrants a Moderate overall risk of bias judgment.
9	Shang et al., 2025	RCT	ROB2	Some concerns	Primarily due to lack of blinding and potential for performance/measurement bias.
10	Singh et. al, 2020	Randomized pilot study	ROBINS I	Critical	The study design and execution are strong for a pilot trial, but the small sample size, dropout imbalance, and challenges in intervention adherence (due to real-world logistics) elevate the overall risk of bias to critical. Findings should inform future trials but not yet guide clinical practice.
11	Kahkoska et al., 2024	RCT	ROB2	Some concerns	Mainly due to the exploratory, non-prespecified nature of the analysis and modeling choices.
12	Krajnc et al., 2023	Observational	ROBINS I	Moderate	While the study is well-conducted and methodologically sound in several domains, the lack of statistical adjustment for confounding variables, especially in a real-world setting involving diverse diabetes profiles, places the overall risk of bias at a moderate level.
13	Barua et al., 2022	Observational	ROBINS I	Moderate	Despite high data integrity and comprehensive reporting, confounding from unmeasured behavioral and physiological factors limits causal interpretations of CGM and PPG discordance. Thus, the overall risk of bias remains Moderate.

## References

[1] Jiang S., Yu T., Di D., Wang Y., Li W. (2024). Worldwide burden and trends of diabetes among people aged 70 years and older, 1990–2019: A systematic analysis for the Global Burden of Disease Study 2019. Diabetes Metab. Res. Rev..

[2] Golchha S., Sondhi S., Gupta S., Goel A. (2024). Frailty in diabetic population: A study from Northern India. Cureus.

[3] Kulkarni P., Babu P.K., Vanmathi A., Ashwini A., Murthy M.R.N. (2022). Relationship between frailty, glycemic control, and nutritional status among the elderly with diabetes mellitus residing in an urban community of Mysuru. J. Life Health.

[4] Charbit J., De Saint-Germain É.D.R., Boland B., Faraji O.Y., Hanon O. (2023). Évaluation gériatrique chez les patients diabétiques âgés. Méd. Mal. Métab..

[5] Wu X., Tang Y., He Y., Wang Q., Wang Y., Qin X. (2024). Prevalence of cognitive impairment and its related factors among Chinese older adults: An analysis based on the 2018 CHARLS data. Front. Public Health.

[6] Shukla A., Shukla A., Misra P. (2023). Prevalence and determinants of cognitive impairment in an elderly population: A hospital-based study. Ann. Geriatr. Educ. Med. Sci..

[7] Rattanawan P. (2022). Correlations between hand dexterity and bimanual coordination on the activities of daily living in older adults with mild cognitive impairment. Dement. Geriatr. Cogn. Disord. Extra.

[8] Davidson S., Learman K., Zimmerman E., Rosenfeldt A.B., Koop M., Alberts J.L. (2024). Older adults are impaired in the release of grip force during a force tracking task. Exp. Brain Res..

[9] Fukumoto Y., Wakisaka T., Misawa K., Hibi M., Suzuki T. (2023). Decreased nerve conduction velocity may be a predictor of fingertip dexterity and subjective complaints. Exp. Brain Res..

[10] Grazziano E.D.S. (2018). Glycemic control of elderly patients with diabetes mellitus undergoing surgery: A mini review. Nurs. Care Open Access J..

[11] Untari E.K., Yuswar M.A. (2024). Hypoglycemia in geriatric patients with diabetes: A review. Pharm. J. Indones..

[12] Umegaki H. (2024). Management of older adults with diabetes mellitus: Perspective from geriatric medicine. J. Diabetes Investig..

[13] Leite S.A.O., Silva M.P., Lavalle A.C.R., Bertogy M.C.V., Bastos M., Kuklik S.C.V., Umpierrez G. (2023). Use of continuous glucose monitoring in insulin-treated older adults with type 2 diabetes. Diabetol. Metab. Syndr..

[14] Singh L.G., Levitt D.L., Satyarengga M., Pinault L., Zhan M., Sorkin J.D. (2020). Continuous glucose monitoring in general wards for prevention of hypoglycemia: Results from the glucose telemetry system pilot study. J. Diabetes Sci. Technol..

[15] Pratley R.E., Kanapka L.G., Rickels M.R., Ahmann A., Aleppo G., Beck R.W. (2020). Effect of continuous glucose monitoring on hypoglycemia in older adults with type 1 diabetes: A randomized clinical trial. JAMA.

[16] Miller K.M., Kanapka L.G., Rickels M.R., Ahmann A.J., Aleppo G., Ang L. (2022). Benefit of continuous glucose monitoring in reducing hypoglycemia is sustained through 12 months of use among older adults with type 1 diabetes. Diabetes Technol. Ther..

[17] Chu C., Li J., Yang X., Zhao H., Wu Z., Xu R. (2024). Continuous glucose monitoring versus conventional glucose monitoring in the ICU: A randomized controlled trial. J. Crit. Care.

[18] Chao C., Andrade S.B., Bergford S., Calhoun P., Welsh J.B., Walker T.C. (2023). Assessing non-adjunctive CGM safety at home and in new markets (ANSHIN). Endocrinol. Diabetes Metab..

[19] Munshi M., Slyne C., Davis D., Michals A., Sifre K., Dewar R. (2022). Use of technology in older adults with type 1 diabetes: Clinical characteristics and glycemic metrics. Diabetes Technol. Ther..

[20] Zelnick L.R., Batacchi Z.O., Ahmad I., Dighe A., Little R.R., Trence D.L. (2020). Continuous glucose monitoring and use of alternative markers to assess glycemia in chronic kidney disease. Diabetes Care.

[21] Toschi E., Slyne C., Sifre K., O’Donnell R., Greenberg J., Atakov-Castillo A. (2020). The relationship between CGM-derived metrics, A1c, and risk of hypoglycemia in older adults with type 1 diabetes. Diabetes Care.

[22] Bosi E., Gregori G., Cruciani C., Irace C., Pozzilli P., Buzzetti R. (2022). The use of flash glucose monitoring significantly improves glycemic control in type 2 diabetes managed with basal bolus insulin therapy compared to self-monitoring of blood glucose: A prospective observational cohort study. Diabetes Res. Clin. Pract..

[23] Shang J., Yuan Z., Zhang Z., Zhou Q., Zou Y., Wang W. (2025). Effectiveness of continuous glucose monitoring on short-term, in-hospital mortality among frail and critically ill patients with COVID-19: Randomized controlled trial. J. Med. Internet Res..

[24] Kahkoska A.R., Shah K.S., Kosorok M.R., Miller K.M., Rickels M., Weinstock R.S. (2024). Estimation of a machine learning-based decision rule to reduce hypoglycemia among older adults with type 1 diabetes: A post hoc analysis of continuous glucose monitoring in the WISDM study. J. Diabetes Sci. Technol..

[25] Krajnc M., Kravos Tramšek N.A. (2023). Glycaemia in low-premixed insulin analogue type 2 diabetes patients in a real-world setting: Are the CGM targets met?. Eur. J. Med. Res..

[26] Barua S., Wierzchowska-Mcnew R.A., Deutz N.E.P., Sabharwal A. (2022). Discordance between postprandial plasma glucose measurement and continuous glucose monitoring. Am. J. Clin. Nutr..

[27] Cuevas-Budhart M.A., Juncos Ríos D.A., Ponce Villavicencio M., Ávila Diaz M., Ilabaca Avendaño M.B., Rocha-Carrillo M.B., Paniagua R. (2025). Patient Experience with Continuous Glucose Monitoring During Dialysis in Type 2 Diabetes: A Qualitative Study. J. Clin. Med..

